# Chitosan-Based Carbon Quantum Dots for Biomedical Applications: Synthesis and Characterization

**DOI:** 10.3390/nano9020274

**Published:** 2019-02-16

**Authors:** Łukasz Janus, Marek Piątkowski, Julia Radwan-Pragłowska, Dariusz Bogdał, Dalibor Matysek

**Affiliations:** 1 Department of Biotechnology and Physical Chemistry, Faculty of Chemical Engineering and Technology, Cracow University of Technology, Cracow 31-155, Poland; mpiatkowski@chemia.pk.edu.pl (M.P.); jrpraglowska@chemia.pk.edu.pl (J.R.-P.); pcbogdal@cyf-kr.edu.pl (D.B.); 2 Department of Geological Engineering, Faculty of Mining and Geology, Technical University of Ostrava, Institute of Clean Technologies for Mining and Utilization of Raw Materials for Energy Use, Ostrava 70800, Czechia; university@vsb.cz

**Keywords:** carbon quantum dots, fluorescent biomaterials, bionanomaterials

## Abstract

Rapid development in medicine and pharmacy has created a need for novel biomaterials with advanced properties such as photoluminescence, biocompability and long-term stability. The following research deals with the preparation of novel types of *N*-doped chitosan-based carbon quantum dots. Nanomaterials were obtained with simultaneous nitrogen-doping using biocompatible amino acids according to Green Chemistry principles. For the carbon quantum dots synthesis chitosan was used as a raw material known for its biocompability. The nanomaterials obtained in the form of lyophilic colloids were characterized by spectroscopic and spectrofluorimetric methods. Their quantum yields were determined. Additionally the cytotoxicity of the prepared bionanomaterials was evaluated by XTT (2,3-Bis-(2-methoxy-4-nitro5-sulfophenyl)-2H-tetrazolium-5-carboxanilide salt) method. Our results confirmed the formation of biocompatible quantum dots with carbon cores exhibiting luminescence in visible range. Performed studies showed that modification with lysine (11.5%) and glutamic acid (7.4%) had a high impact on quantum yield, whereas functionalization with amino acids rich in S and N atoms did not significantly increase in fluorescence properties. XTT assays as well as morphological studies on human dermal fibroblasts confirmed the lack of cytotoxicity of the prepared bionanomaterials. The study shows chitosan-based quantum dots to be promising for biomedical applications such as cell labelling, diagnostics or controlled drug delivery and release systems.

## 1. Introduction

Rapid development of medicine as well as increasing numbers of patients suffering from cancer causes a need for new types of biomaterials with advanced properties. Special attention is focused on materials with fluorescent properties which can be applied as powerful diagnostic tools as well as elements of controlled drug delivery and release systems. Some of them can also act as theragnostic agents or as cell-labelling agents. Although numerous fluorescent dyes have already been developed, there is still a lack of biomaterials which are non-cytotoxic, water soluble and characterized by strong photoluminescence and reasonable stability [1,2,3,4].

One of the most promising nanomaterials are quantum dots (QD)s which can be prepared from various sources. QDs are nanoparticles of quasi-spherical shape with a size below 10 nm and luminescent properties [5,6]. QDs with metallic or semi-metallic cores can be toxic. In some cases QDs may lose their luminesce properties (photobleaching) or can change from a fluorescent state to a non-fluorescent state temporarily (photoblinking). Therefore, currently scientists are working on carbon-based QDs which can be applied in medicine and pharmacy due to their good biocompability as well as water-dispersity [2,5]. Carbon quantum dots (CQDs), unlike other C-based materials such as carbon nanotubes, graphenes, fullerenes, nanodiamonds and carbon nanohorns, are much simpler and cheaper to produce and do not exhibit cytotoxicity. They can be described as a carbogenic cores with surface functional groups. Usually, they consist of amorphous to a nanocrystalline cores with sp2 carbon. CQDs exhibit polar nature and can be easily modified due to the presence of various functional groups on their surfaces such as hydroxyl, carboxyl, ester, ether or amino. Most importantly, carbon quantum dots can be prepared according to Green Chemistry principles without using toxic reagents [7,8]. As raw materials, almost any kind of bio-waste rich in C atoms can be applied, such as tea leaves, coffee grounds or fruit peels. The replacement of toxic reactants with natural substrates helps to obtain biocompatible CQDs. Thus, they can be described as materials with very high potential in biomedical applications.

Carbon quantum dots are prone to chemical and physical modifications with inorganic, organic polymeric or biological species. What is important, their photoluminescent properties are not only size- and shape-dependent. Edge shapes, surface ligands and defects also play significant roles in determining their final characteristics. Another extra-ordinary feature of CQDs is that their photoluminescence depends on excitation wavelength. Thus, when CQDs are excited by UV to VIS-their emission wavelengths range from the UV to the near-IR region [9,10,11]. 

CQDs can be prepared by two general methods. The first one is called top-down. The second one is named bottom-up. In this method CQDs are obtained as a result of the carbonization process. Top-down methods are harsher as these require high temperatures and pressures [12]. As raw materials graphite powder or multi-walled carbon nanotubes can be used. The best-known top-down methods include arc-discharge, laser ablation and electrochemical methods. Nevertheless, the purification and functionalization of CQDs involves complicated and time-consuming steps, which makes them less interesting, both from a scientific and commercial point of view [13].

On the contrary, bottom-up methods are known to be much more eco-friendly as these often use as raw materials small biomolecules such as fructose or glucose rich in carbon atoms. These methods require application of external sources of energy including microwave heating, ultrasounds or conventional heating. For instance, microwave-assisted conditions enable very fast preparation of CQDs using polyhydroxy carbonates and some functionalizing agents in less than 10 min [8]. It is known, that functionalization of the carbon quantum dots enhances their photoluminescence properties which enables the preparation of the nanomaterials with tailored properties. Such functionalization can be achieved by controlling their surface states. One of the most common methods is implementation of the amino groups through passivation reactions. Numerous reports suggest that general mechanism of the passivation using agents involve terminal-NH_2_ groups filling the surface defects of the CQDs. Another way of quantum yield improvement is doping of the CQDs with nitrogen atoms. Nevertheless, scientists are still facing problems with the preparation of carbon quantum dots which are not only biocompatible and non-cytotoxic but also characterized by good photostability and high quantum yield [8,14,15,16]. 

Chitosan is a biopolymer obtained from chitin by deacetylation reactions. It contains amino glucose and *N*-acetylaminoglucose mers linked by glycosidic bonds. Due to its biocompability, biodegradability as well as its lack of cytotoxicity it is widely applied in medicine and pharmacy as a three-dimensional scaffold in wound dressings or elements of controlled drug delivery and release systems. Since this polymer is not only rich in C atoms but it also contains numerous -OH and-NH_2_ groups which make it an interesting raw material for CQDs preparation for biomedical applications.

Until now, there have been numerus attempts at modifying CQDs. Nevertheless, simple and controllable functionalization and doping of CQDs are still crucial issues, especially in the field of life sciences, where very good photoluminescence properties must be followed by the lack of cytotoxicity [10,14,17]. The application of microwave radiation enables generation of high temperatures in very short periods of time due to the local hot points and high intensity of radiation. Aforementioned heating method enables crosslinking of various polymers monomers due to high local temperatures which may be followed by the carbonization [18]. Therefore, the use of microwave field leads to the fast obtainment of CQDs which would not be created under conventional heating. However, the efficiency of microwave fields is correlated with the ability of mixture components to absorb this kind of radiation. There are reports of CQDs preparation using monomers containing COOH and NH_2_ groups. Nevertheless, the application of compounds such as diethylamine or tris(2-aminoethyl)amine may lead to the cytotoxicity of the prepared nanomaterials [18,19,20,21,22,23,24,25,26,27,28].

The aim of the following research was to obtain a new kind of chitosan-based carbon quantum dots according to Green Chemistry principles with low cytotoxicity that could be used to prepare biomaterials. Although there are some papers considering application of chitosan as a raw material, none of them reports functionalization using amino acids in the field microwave radiation [23,24,25]. It this paper, a novel type of CQDs from chitosan was prepared by means of microwave processing. CQDs were simultaneously functionalized by functional groups containing nitrogen applying biocompatible compounds naturally occurring in human body. The obtained nanomaterials were characterized by UV-VIS spectroscopy and spectro-fluorimetry. The spectra confirmed preparation of two kinds of nanomaterials including nanodots and low-molecular fluorescent by-products. Moreover, quantum yield was determined and proved very good photoluminescence properties of the CQDs. Additionally, cytotoxicity of the chitosan CQDs was investigated confirming biocompability of the prepared nanodots.

## 2. Materials and Methods

### 2.1. Materials

Chitosan was purchased from Vanson, Everett, WA, USA (300,000 g/mol). Sodium hydroxide, lysine, glutamic acid, cysteine, hydrochloric acid were purchased from Sigma Aldrich, Poznań, Poland. Casein hydrolysate was prepared from natural casein as a result of the mineralization reaction with hydrochloric acid under microwave-assisted conditions. XTT (2,3-Bis-(2-Methoxy-4-Nitro-5-Sulfophenyl)-2H-Tetrazolium-5-Carboxanilide) assay, human dermal fibroblasts (HDF), fibroblast growth medium (FGM) were purchased from Sigma Aldrich, Poland. Multi-hole plates were purchased from Nest, Gdańsk, Poland. 

### 2.2. Methods

#### 2.2.1. Chitosan-Based Carbon Quantum Dots Synthesis and Functionalization

Chitosan-based CQDs were prepared in microwave radiation fields using a Prolabo Synthwave 402 reactor according to data given in Table 1. CQDs were simultaneously functionalized by N-doping with amino acids and casein hydrolysate as modifying agents. Briefly, for the CQDs synthesis and modification chitosan (0.25 g), HCl (0.10 mL) and appropriate functionalizing agents were placed into the reaction vessel and subjected to microwave radiation (300 W). 

The post-reaction mixture was diluted with distilled water and treated in an ultrasonic bath for 10 min. Next, the mixture was filtrated on filter paper to remove large particles. Thereafter, the mixture was neutralized to pH = 7 using NaOH solution and filtrated by membrane filters (0.22 µm). In the next step, the obtained CQDs were purified using dialysis tubing (MWCO 500-1000) method to separate nanomaterials from other by-products such as low molecular weight molecules. Dialysis was conducted for 4 days. The solvent was exchanged daily. The prepared solutions were diluted with distilled water and used for the experiments.

#### 2.2.2. Fourier Transform Infrared Spectroscopy (FT-IR) Analysis

All FT-IR analyses were carried out using IR Nicolet 6700 Thermo Scientific spectrometer (USA). For the analysis the samples were evaporated and placed in KBr pellet. The range was between 400 and 4000 cm^−1^ with 32 scans and the resolution was 4 cm^−1^.

#### 2.2.3. Dynamic Light Scattering (DLS) Analysis

All DLS analyses were performed using Zeta-Sizer Nano.

#### 2.2.4. Scanning Electron Microscopy (SEM) Analysis

All SEM analyses were performed using FEI QUANTA 650 FEG. Microphotographs were taken under the pressure of 50 Pa and HV of 10.00 kV.

#### 2.2.5. Chitosan-Based Carbon Quantum Dot Spectroscopic Characterization

For the measurements an Aligent 8453 spectrophotometer was used. Spectra of the perpmeates obtained during the dialysis were measured. 

#### 2.2.6. Chitosan-Based Caron Quantum Dot Spectrofluorimetric Characterization

Fluorescence measurements were performed using an Ocean Optics 2000 spectro-fluorimeter for non-diluted CQD solutions. Measurements were conducted using cuvettes with an optical length of 1 cm with the integration time of 10,000 ms. Fluorescence excitation was performed with an UV light-emitting diode with an emission maximum at 365 nm using distilled water as a blank probe.

#### 2.2.7. Quantum Yield Determination of Chitosan-Based Carbon Quantum Dots

The quantum yield of the obtained products was determined according to Equation (1):

(1) $$Q_{s}=Q_{r}\left(\frac{A_{r}}{A_{s}}\right)\left(\frac{E_{s}}{E_{r}}\right){\left(\frac{\eta_{s}}{\eta_{r}}\right)}^{2},$$

*Q* = Fluorescence quantum yield; *ŋ* = Refractive index of the solvent; *A* = Absorbance of the solution; *E* = Integrated fluorescence intensity of emitted light; Subscripts ‘*r*’ and ‘*s*’ refer to the reference and unknown fluorophore respectively.

The fluorescence quantum yield was determined by comparison to quinine sulphate (dissolved in 0.1 M sulphuric acid solution) standard solutions, which exhibited a fluorescence quantum yield of 0.54 at 365 nm.

#### 2.2.8. Cytotoxicity of the Prepared Chitosan-Based CQDs

Cytotoxicity of the prepared CQDs was investigated by a fast and sensitive XTT assay method. Experiments were carried out using primary cells - human dermal fibroblasts (HDF). XTT test enable determination of the cell viability by enzymatic reactions in which viable cells convert water-soluble XTT salt into a water-soluble, orange formazan product. For the experiments all vessels were sterilized using an autoclave. HDF were cultured in ready to use complete fibroblast growth medium. The medium was changed every two days. Cell culture was performed for 5 days. HDF were cultured at 37 °C, 5% CO_2_ and 95% humidity. For the CQDs testing 96-hole plates were used. The cell culture with CQDs solutions lasted 48 h. Each experiment was repeated 5 times. The morphology of the cultured cells was investigated using an inverted microscope under 40× magnification.

## 3. Results and Discussion

### 3.1. FT-IR Analysis of Chitosan-Based CQDs

**Figure 1:**
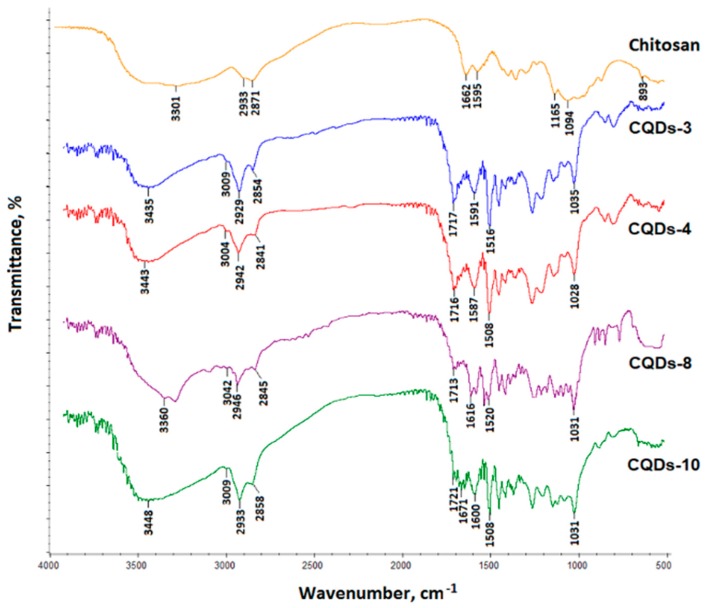
FT-IR spectra of the pure chitosan and CQD samples.

For the chemical structure of the prepared CQDs verification, the FT-IR analysis was carried out (Figure 1). FT-IR spectra present pure chitosan as well as CQD-3, CQD-4, CQD-9 and CQD-10 samples (the CQDs with the highest quantum yield). Pure chitosan spectrum exhibits a band typical for free hydroxyl groups at 3301 cm^−1^ coming from *N*-acetylaminoglucose and amino glucose mers. A band characteristic for aliphatic groups is visible at 2933 cm^−1^ and 2871 cm^−1^. A band with the maximum at 1662 cm^−1^ confirms the presence of *N*-acetylated units. Moreover, bands typical for free NH_2_ groups coming from deacetylated units are visible at 1595 cm^−1^ and 1165 cm^−1^. Finally, a band typical for glycosidic bonds between chitosan mers at 1094 cm^−1^ as well as a band coming from pyranose ring at 893 cm^−1^ can be observed. The FT-IR spectra of CQDs-3, CQDs-4, CQDs-8 and CQDs-10 are significantly different from pure chitosan spectrum which confirms polymer transformation into carbon quantum dots. It can be noticed, that bands typical for chitosan chain structure coming from pyranose rings and glycosidic bonds have disappeared. At the same time, some new bands typical for aromatic structures have appeared (CQDs-3- 1516 cm^−1^, CQDs-4- 1508 cm^−1^, CQDs-8- 1520 cm^−1^, CQDs-10- 1508 cm^−1^ respectively). The bands characteristic for unsubstituted C=C bonds have arisen at CQDs-3- 3009 cm^−1^, CQDs-4- 3004 cm^−1^, CQDs-8- 3042 cm^−1^, CQDs-10- 3009 cm^−1^ respectively. On the other hand, bands typical for hydroxyl groups are still present (CQDs-3- 3435 cm^−1^, CQDs-4- 3443 cm^−1^, CQDs-8- 3360 cm^−1^, CQDs-10- 3448 cm^−1^ respectively) and so do bands coming from –CH_2_– groups (CQDs-3- 2929 and 2854 cm^−1^, CQDs-4- 2942 and 2841 cm^−1^, CQDs-8- 2946 and 2845 cm^−1^, CQDs-10- 2933 and 2858 cm^−1^ respectively). An increased intensity of the bands typical for free amino groups can be observed (CQDs-3- 1591 cm^−1^, CQDs-4- 1587 cm^−1^, CQDs-8- 1616 cm^−1^, CQDs-10- 1600 cm^−1^ respectively) which confirms the *N*-doping of the carbon core. The spectra also exhibit bands typical for free carboxyl groups (CQDs-3- 1717 cm^−1^, CQDs-4- 1716 cm^−1^, CQDs-8- 1713 cm^−1^, CQDs-10- 1721 cm^−1^ respectively) which have arisen as a result of the modifying agents (amino acids) thermal degradation. The presence of aforementioned functional groups of polar nature (NH_2_, OH, COOH) is responsible for water-solubility of the carbon quantum dots [18,28].

### 3.2. Spectroscopic Studies on Chitosan-Based CQDs

Carbon quantum dots mainly feature two absorption bands: a π-π* electron transfer band which appears when double C=C bonds are present in the carbon skeleton and a n-π* electron transfer which arises when C=O groups are present. What is important, intensity of these bands is variable. In the first kind of CQDs the bands are intense and the peaks visibly stand out from the base-line spectrum; in the other case, these bands are almost invisible. Carbon quantum dots have spherical structure and are built up from sheets of carbon atoms combined in the crystalline structure. Absorption properties which are correlated with fluorescence are caused by a disturbance of the graphite crystalline structure as a result the incorporation of N and O atoms. Higher crystalline structure disorder and number of chromophores chemically bonded with carbon core leads to the improvement of absorption properties of the nanoparticles. The disturbance of the crystalline structure leads to the increased possibility of the appearance of the additional absorption bands coming not only from the carbon core but also attached particles modifying CQDs structure [18,19,20,21,22,23,24]. 

**Figure 2:**
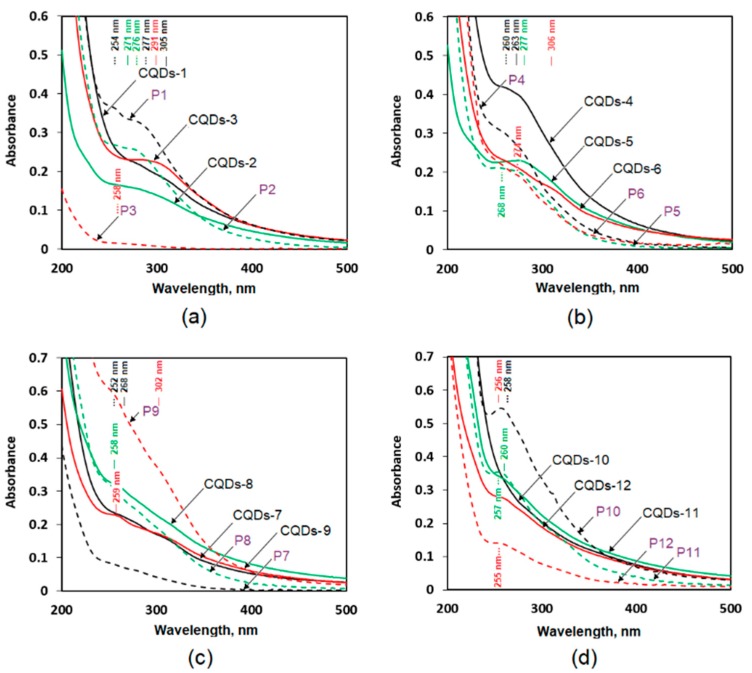
UV-Vis spectra of the obtained CQDs and their permeates (**a**) CQDs1-3 samples and their permeates, (**b**) CQDs4-6 samples and their permeates, (**c**) CQDs7-9 samples and their permeates and (**d**) CQDs-10-11 samples.

Figure 2 presents UV-VIS spectra of the prepared CQDs as well as permeates obtained during nanomaterials purification from low-molecular-weights side-products. Spectra given in panel (a) refer to carbon nanoparticles prepared from chitosan using lysine as a modifying agent and by applying increasing carbonization times under microwave irradiation. It can be noticed that the shorter carbonization time (5–3 min), the stronger intensity of the absorption band of the generated CQDs.

One can notice, that CQDs-1 samples do not feature any clear bands, while in CQD-2 samples an absorption band appears at 271 nm. In the CQD-3 samples, an absorption band is visible at 291 nm. The appearance of the strong absorption band suggests the formation of a chromophore system which emits strong fluorescence as a result of radiation deactivation [18,19,20,21,22,23,24,25]. Figure 2 shows that all permeates (P1-P3) have different absorption characteristics which confirms the presence of two fractions of products during carbonization process. Moreover, it proves that the obtained CQDs were completely purified. The spectra given in the panel (b) were observed on CQDs obtained during chitosan carbonization in the presence of glutamic acid. These are completely different from those of the previous samples (CQDs1-3). A lowering of the reaction time caused the disappearance of the strong absorption band visible at 263 nm (CQDs-4). In the case of CQDs-5 samples this band has shifted to 277 nm and it has almost completely disappeared in samples CQD-6. Based on the spectra of P4-P6 permeates, it can be concluded that both CQD-4-6 samples as well as P4-P6 permeates had similar chromophore systems in their structure, since their bands had similar shape. Panel (c) presents absorption spectra for CQDs prepared from chitosan functionalized with cysteine—an amino acid containing sulphur and nitrogen atoms. These spectra do not feature strong bands with the exception of the CQD-9 sample which was carbonized for 3 min and which features two clear bands at 259 nm and 302 nm. At the same time, one can notice that the permeate of this sample (P9) exhibited the most pronounced difference in the ability of radiation absorption.

To determine the impact of amino acid functionalization on chitosan CQD properties, CQDs were also modified with casein hydrolysate. The hydrolysate was dried and added to the reaction mixture. Panel (d) shows the absorption bands of the obtained samples and their permeates. It can be noticed that a strong absorption band is present only in the case of CQD-12 samples at 256 nm. On the other hand, permeates P10-P12 exhibited strong absorption bands at 255–258 nm which suggests that conjugated systems of N=N bonds in the low-molecular products were created. The obtained spectra correlate with the data of other researchers, who obtained chitosan-based nanomaterials. Nevertheless, the intensity of absorption bands may be misleading in the case of not fully purified CQDs [23,24].

### 3.3. Fluorescence Studies on Chitosan-Based CQDs

**Figure 3:**
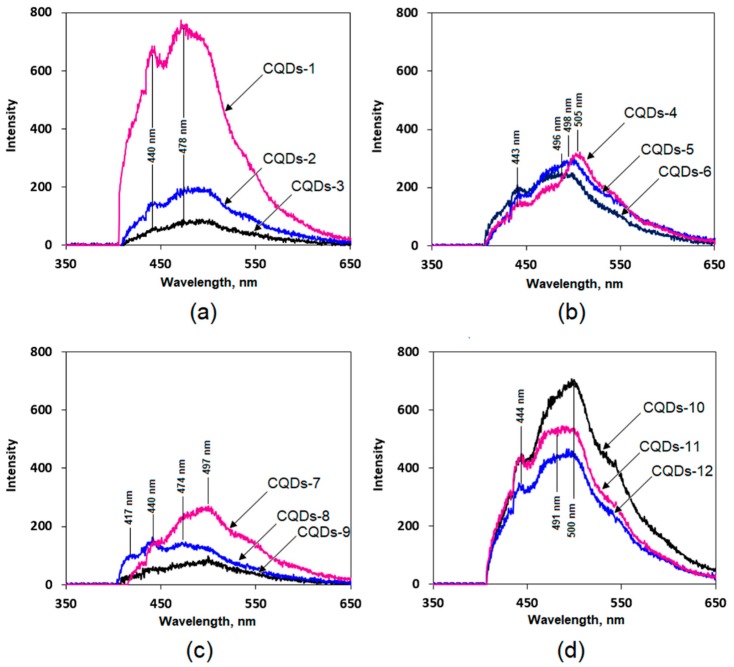
Fluorescent Spectra of the Obtained Chitosan-Based CQDs (**a**) CQDs1-3 samples, (**b**) CQDs4-6 samples, (**c**) CQDs7-9 samples and (**d**) CQDs-10-11 samples.

Figure 3 presents fluorescence spectra of the prepared samples. Panel (a) shows fluorescence spectra observed on primary CQDs solutions diluted to 50 mL volume using distilled water. It can be noticed, that an increase in carbonization duration leads to the formation of larger amounts of CQDs, which can be confirmed by the strong fluorescence emission intensity of the CQDs-1 sample. In the all three cases (CQDs1-3) similar radiation emission maxima can be noticed at 440 nm and 478 nm. On this basis, it can be concluded that although the carbonization time has an impact on the number density of CQDs, it does not influence their photoluminescence properties. In the case of samples modified with glutamic acid (panel (b)) it can be observed that their luminescence properties are highly dependent on the carbonization reaction duration. By applying longer synthesis times as well as higher carbonization temperatures, quantum dots with emission maxima shifted to longer wavelengths are obtained. It can be observed, that a decrease in the carbonization duration fluorescence emission maxima shift from 525 nm through 498 nm up to 496 nm. Thus, it can be concluded that CQDs with different luminescent characteristics are obtained. Nanomaterials which emit radiation at an excitation wavelength of 365 nm yield higher emission intensity in the yellow region of the spectrum (CQDs-4). All three samples (CQDs4-6) show similar fluorescence emission intensities. Carbon quantum dots prepared from chitosan modified with cysteine (panel (c)) exhibit a strong correlation of fluorescence emission maxima with the carbonization duration. In the case of samples carbonized for 5 min (CQDs-7) as well as for 3 min (CQDs-9) it can be noticed that the emission maxima have shifted to a higher wavelength of 497 nm. When the duration time is shortened nanomaterials with blue fluorescence are obtained. What is interesting, CQD-8 samples exhibit two emission fluorescence bands in the region of lower wavelengths at 417 nm and 440 nm respectively. The samples prepared from casein hydrolysate (CQDs-10-12) have almost identical fluorescence spectra which do not depend on the carbonization duration. However, they differ in the fluoresce intensity. In the case of the CQDs-10-12 samples three characteristic bands at 440 nm, 491 nm and 500 nm are visible. The obtained fluorescence spectra are typical for carbon quantum dots and correspond to the other researchers results [18,23,24,25,28]. 

### 3.4. Quantum Yield, Size and Morphology of Chitosan-Based CQDs

For the determination of the quantum yield of the prepared bionanomaterials, a comparative method was applied with quinine sulphate solutions (0.1 M) in sulphuric acid serving as a reference medium. During measurements standard solutions were prepared as well as samples of different concentrations (0–0.100 A (Absorbance)). The results are given in Table 2.

The results show that the highest fluorescence quantum yield was noticed in the case of the sample prepared from chitosan and functionalized by lysine (CQDs-3) and carbonized for 3 min in the microwave radiation field. The quantum yield for this sample is 11.5%. Another sample with very good quantum yield (7.4%) was obtained through chitosan carbonization with glutamic acid as a modifying agent (CQDs-4). In this second case a long carbonization time was applied (5 min). The quantum yields are higher than in the case of CQDs prepared by other researchers (4.34%) using chitosan under harsher conditions without any additional modifying agents (300 °C, 2 h) [23]. There are reports considering obtainment of chitosan nanomaterials by hydrothermal method (180 °C, 12 h) of better fluorescence properties, however the authors did not perform dialysis process. Thus, it was not confirmed that high fluorescence yield can be assign to CQDs, only high- and low-molecular fluorescent compounds [24]. Interestingly, the lowest quantum yields were obtained for carbon quantum dots modified with cysteine as well as with amino acid/peptide mixtures (casein hydrolysate). These results are unexpected, since it was expected that the introduction of a high amount of S and N atoms into the reaction mixture will result in a higher quantum yield due to the introduction of the new chromophore systems containing sulphur atoms. Moreover, the application of various amino acids with different chemical structure did not result in the expected formation of numerous chromophore systems with high fluorescence intensity. This effect is quite surprising, since cyclic and aromatic amino acids such as proline, phenylalanine, tyrosine or tryptophan were used. Overall, it can be concluded that the application of isolated amino acids containing two nitrogen atoms (lysine) gave the best results and lysine can be considered as the best functionalizing agent. The quantum yields can be correlated with the size of the obtained nanoparticles (Table 2). 

**Figure 4:**
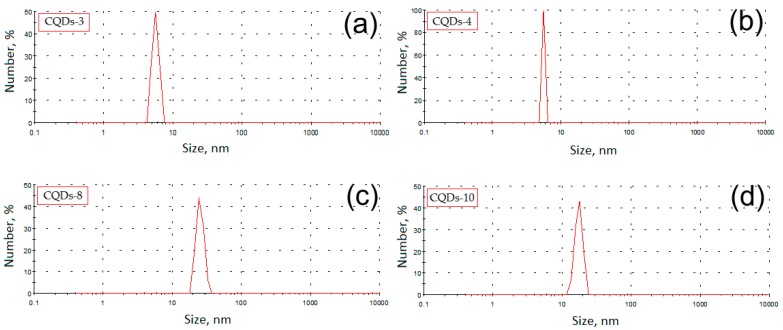
Size distribution of the (**a**) CQD-3, (**b**) CQD-4, (**c**) CQD-8 and (**d**) CQD-10 samples.

**Figure 5:**
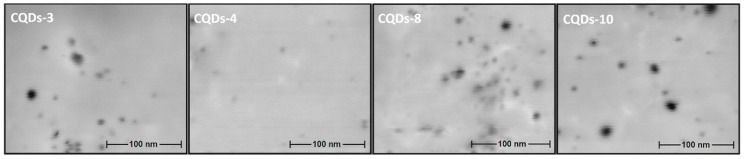
SEM microphotographs of the CQD-3, CQD-4, CQD-8 and CQD-10 samples.

Figure 4 presents results of DLS analysis for the samples with the highest fluorescence quantum yield. It can be noticed that the fluorescence properties are hardly correlated with the nanoparticles size. Carbon dots diameters are corresponding to the carbonization time. It may be assumed, that the longer reaction duration caused more intense degradation of the polymeric chains followed by formation of the smaller particles. The other factor affecting nanoparticles sizes is the type of modifying agent which was applied. The application of simpler functionalizers enabled preparation of smaller particles. It correlates with the results of other researchers, who obtained nanodots of size below 9 nm from chitosan using acetic acid as only additional component [24]. It may be assumed, that modifying substances could vary in the ability of microwave radiation absorption thus affecting the mechanism of carbon quantum dots formation. Figure 5 presences microphotographs of the CQDs with the highest fluorescence quantum yield. It can be noticed that all of the nanoproducts have spherical shape and their size is correlated with DLS results. The morphology as well as size of the chitosan nanomaterials correlates with other researchers data. Therefore, it can be stated that prepared nanomaterials are confirmed to be carbon quantum dots [18,23,24,25,26,27,28]. 

**Figure 6:**
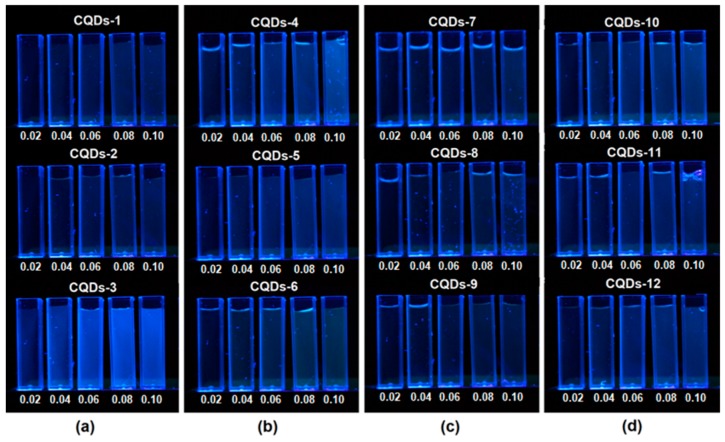
Chitosan-based CQDs under UV radiation (**a**) CQDs1-3 samples, (**b**) CQDs4-6 samples, (**c**) CQDs7-9 samples and (**d**) CQDs-10-11 samples.

Figure 6 presents solutions of chitosan-based quantum dots with different concentrations leading to absorbances of A = 0.02; 0.04; 0.06; 0.08; 0.10 respectively. The samples were kept in darkness. For fluorescence excitation a mercury lamp with an emission maximum of 365 nm was used. The figure shows that the highest fluorescence intensity exhibit CQD-3 and CQD-4 samples.

### 3.5. Cytotoxicity Study

**Figure 7:**
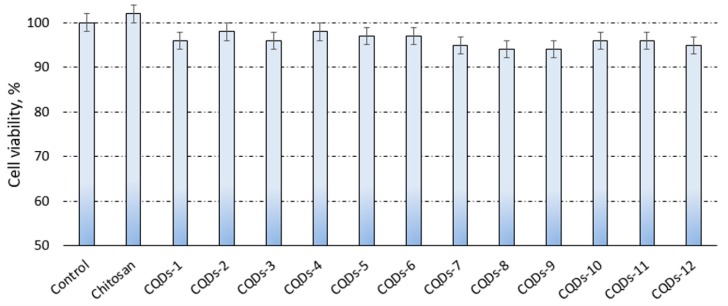
XTT assay study on human dermal fibroblasts.

**Figure 8:**
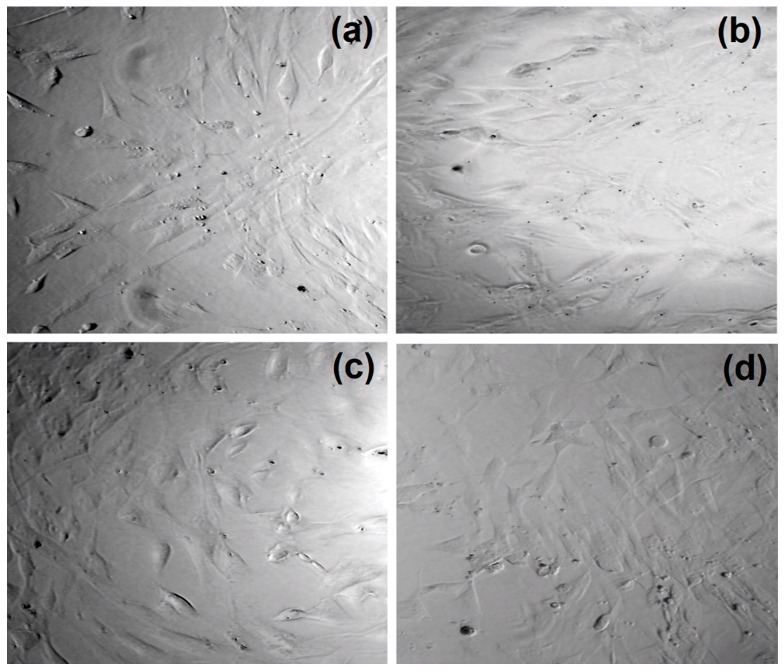
Microphotographs of human dermal fibroblasts cultured with CQDs after 48 h, (**a**) CQDs-3, (**b**) CQDs-4, (**c**) CQDs-12, (**d**) control.

Figure 7 presents the results of the XTT assay. It can be noticed that all evaluated samples were non-cytotoxic, since cell viability was no less than 94% as compared to the control sample–cells cultured at standard conditions as well as the raw chitosan (102%). Such results suggest that although nanomaterials may penetrate cell membranes these do not affect in any significant manner cell cycles such as glycolysis [18]. It can be noticed that the differences in the cell viability hardly depend on the functionalization agents of the CQDs. The lack of cytotoxicity can be explained by the application of non-toxic raw materials as well as efficient purification from unreacted residues. As reported, carbon-based quantum dots are not toxic to cells, however their functionalization may negatively affect this property [18,23]. The application of biocompatible functionalizing agents and the introduction of cells-friendly -NH_2_ groups resulted in the preparation of nanomaterials suitable for biomedical applications. Figure 8 presents microphotographs of human dermal fibroblasts after 48 h culture in the presence of these samples. It can be noticed that all cells are flattened and have standard morphology. Cultured fibroblasts are of typical spindle-like shape. No granules in the cytoplasm can be observed. The cells nuclei are flat and oval. Therefore, it can be stated that the presence of carbon quantum dots does not have any negative impact on the cultured cells. Overall, all investigated nanomaterials can be ascribed as being not cytotoxic and biocompatible [18,23,26,27,28].

## 4. Conclusions

The aim of this study was to develop novel type of nanobiomaterials for biomedical applications with advanced properties using only biocompatible components. A successful attempt was made to obtain chitosan-based carbon quantum dots in a fast and efficient manner according to Green Chemistry principles. Chitosan QCDs were prepared with simultaneous functionalization using amino acids under microwave-assisted conditions which resulted in the preparation of carbon nanodots with very good photoluminescence properties. The microwave radiation enabled chitosan crosslinking followed by carbonization. The research showed that the best modifying agent is lysine which enabled *N*-doping reactions resulting in a high quantum yield of the nanoparticles. The nanoparticles had spherical shape typical for CQDs. Moreover, perfumed XTT test as well as morphological studies on human dermal fibroblasts confirmed the lack of cytotoxicity of the prepared biomaterials. Overall, it can be stated that the proposed synthesis pathway enabled obtaining chitosan-based quantum dots with a high potential in biomedical applications such as cell labelling, diagnostics, theragnostics as well as in controlled drug delivery systems. 

## Figures and Tables

**Table 1:** Chitosan-based CQD synthesis and functionalization parameters.

Sample	Time, min	Functionalizer, g
CQDs-1	5	Lysine, 0.05
CQDs-2	4	Lysine, 0.05
CQDs-3	3	Lysine, 0.05
CQDs-4	5	Glutamic acid, 0.05
CQDs-5	4	Glutamic acid, 0.05
CQDs-6	3	Glutamic acid, 0.05
CQDs-7	5	Cysteine, 0.05
CQDs-8	4	Cysteine, 0.05
CQDs-9	3	Cysteine, 0.05
CQDs-10	5	Casein hydrolysate, 0.05
CQDs-11	4	Casein hydrolysate, 0.05
CQDs-12	3	Casein hydrolysate, 0.05

**Table 2:** Chitosan-based CQDs fluorescence quantum yield results.

Sample	Fluorescence Quantum Yield, %	Particle Diameter, nm
CQDs-1	3.6	5.0
CQDs-2	3.8	5.5
CQDs-3	11.5	5.5
CQDs-4	7.4	5.5
CQDs-5	6.5	6.5
CQDs-6	4.4	8.0
CQDs-7	4.3	8.5
CQDs-8	4.7	11.5
CQDs-9	2.5	12.0
CQDs-10	6.6	11.0
CQDs-11	2.3	12.5
CQDs-12	4.6	14.0
